# Long-Term Changes in Cognition and Physiology after Low-Dose ^16^O Irradiation

**DOI:** 10.3390/ijms20010188

**Published:** 2019-01-07

**Authors:** Alexis Howe, Frederico Kiffer, Tyler C. Alexander, Vijayalakshmi Sridharan, Jing Wang, Fabio Ntagwabira, Analiz Rodriguez, Marjan Boerma, Antiño R. Allen

**Affiliations:** 1 Division of Radiation Health, University of Arkansas for Medical Sciences, Little Rock, AR 72205, USA; ahowe3@jhu.edu (A.H.); FCKiffer@uams.edu (F.K.); Tyler.alexander@stjude.org (T.C.A.); VMohanseenivasan@uams.edu (V.S.); Jwang2@uams.edu (J.W.); MBoerma@uams.edu (M.B.); 2 Department of Pharmaceutical Sciences, University of Arkansas for Medical Sciences, Little Rock, AR 72205, USA; 3 Neurobiology & Developmental Sciences, University of Arkansas for Medical Sciences, Little Rock, AR 72205, USA; FNtagwabira@uams.edu; 4 Department of Neurosurgery, University of Arkansas for Medical Sciences, Little Rock, AR 72205, USA; ARodriguez@uams.edu

**Keywords:** cognition, hippocampus, dendritic spines

## Abstract

Astronauts traveling to Mars will be exposed to high levels of ionizing radiation upon leaving low-Earth orbit. During prolonged space travel, astronauts are exposed to galactic cosmic rays (GCRs) composed of protons; oxygen molecules; and high energy, high mass charged particles. Notably, oxygen molecules can travel through the shielding of spacecraft, potentially impacting 25% of the hippocampus. The aim of the current study was to assess whether ^16^O-particle radiation induced a behavioral deficit and histological changes in mice. Mice were sent to the National Aeronautics and Space Administration (NASA) Space Radiation Laboratory at Brookhaven National Laboratory and exposed to particulate ^16^O radiation at doses of 0 and 0.05 Gy. Nine months after irradiation, the mice were tested for novel object recognition and in the Y-maze, after which the animals were sacrificed. The brains were then dissected along the midsagittal plane for Golgi staining. Exposure to 0.05 Gy significantly impaired novel object recognition. However, short term memory and exploratory activity in the Y-maze were not affected. Micromorphometric analysis revealed significant decreases in mushroom spine density in the dentate gyrus and cornu Ammonis-1 and -3 of the hippocampus. Sholl analysis revealed a significant decrease in dendritic complexity in the dentate gyrus. The present data provide evidence that space radiation has deleterious effects on mature neurons associated with hippocampal learning and memory.

## 1. Introduction

National Aeronautics and Space Administration (NASA) aims to launch a manned mission to Mars during the 2030s—a mission that will help advance our society socially, politically, and technologically [1]. To stay within this range of acceptable risk, NASA must ensure that astronauts will be exposed to the lowest doses of radiation possible, by taking precautions such as meticulously planning each phase of a mission. Working within this level of acceptable risk also ensures that space travel carries approximately the same risk as more traditional professions [2]. Deep-space travel is hazardous to astronauts because it exposes them to ionizing radiation in the forms of galactic cosmic rays (GCRs) and solar energetic particle events (SPEs).

NASA’s current Mars Design Reference Architecture addendum indicates two possible orbital strategies beginning in the mid-2030s, one that ranges a round trip from 560–850 days, and one that ranges from 900–1100 days [3]. The NASA-defined effective whole-body radiation dose equivalent for a 1100 day mission is approximately 900 mSv; however, the true dosage is dependent upon solar cycle activity [4]. Upon leaving low-Earth orbit, astronauts exit the natural layer of protective shielding provided by the atmosphere [5]. GCRs consist of 98% protons and 2% electrons and positrons, and approximately 88% of GCRs consist of ions with an atomic number greater than 2 [6]. ^16^O is especially problematic for astronauts because it can penetrate the shielding of spacecraft, making it the species that astronauts come into contact with most often. Furthermore, coronal mass ejections emit SPEs, made up of particles such as protons and electrons, through space at high speeds. SPEs are particularly dangerous to astronauts because they occur randomly and can contain a large number of particles [7].

Studies of how the central nervous system (CNS) responds to radiation have documented a number of morphological and physiological consequences of such exposure [8,9,10]. Exposure to high linear energy transfer (LET) radiation (>1 Gy) leads to short- and long-term deficits in hippocampal-dependent spatial learning [11,12,13]. Recent rodent studies have demonstrated numerous behavioral deficits as a result of ^16^O exposures at deep-space-relevant energies. Rats who received doses of 0.01 or 0.05 Gy (1000 MeV/n) at 15 months of age showed anxiety via the Elevated Plus maze [14]. Mice who received 0.3 Gy (600 MeV/n) at 6 months failed to discriminate the novel object and location via the Novel Object Recognition (NOR) and Object in Place paradigms [15]. Seven-week-old rats who received 0.01 or 0.05 Gy (600 MeV/n) failed to discriminate the novel object in the NOR [16,17]. We have recently shown deficits in short-term memory via the Y-maze as a result of exposure to 0.1 and 0.25 Gy ^16^O (600 MeV/n) in 6-month old mice two weeks post exposure [18].

Cognitive decline following gamma irradiation can also manifest as decreased working memory, cognitive control and flexibility [19]. Progressive hippocampus-dependent learning dysfunction has been observed in mice at 1 and 5 months after 20 Gy fractionated cranial irradiation [20]. Studies have also documented gamma-induced decreases in dendritic spine density and changes in spine morphology in hippocampal neurons. In a study by Chakraborti et al., we used 2 months old male mice to measure negative effects of 10 Gy of cranial radiation on hippocampal spine density and morphology in the hippocampus. We found that the spine morphology and density of the hippocampus was greatly affected by cranial radiation [21]. Gamma radiation has been shown to significantly compromise neuronal morphology in the hippocampus. After 10 Gy of cranial irradiation, Parihar et al. observed significant decreases in dendritic complexity, dendritic branching, area and length in irradiated mice [22]. The present study was conducted in order to evaluate long term the effects ^16^O radiation on cognition, spine densities, dendrite complexity as well as the mRNA expression of glutamate receptors in the hippocampus.

## 2. Results

### 2.1. Behavior

**Figure 1:**
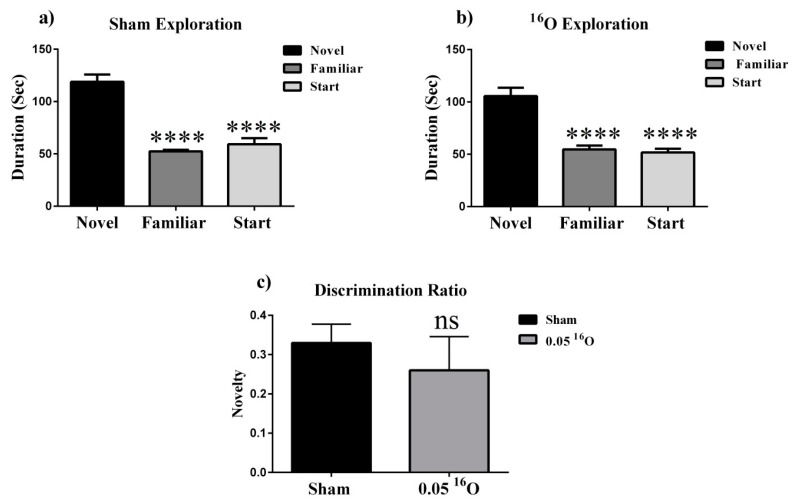
Y-maze. (**a**–**c**) All treatment groups spent significantly more time exploring the novel arm during the testing phase of the Y-maze, indicating no short-term memory deficits. Average ± standard error of the mean (SEM) (*n* = 12); **** *p* < 0.001.

We used the Y-maze to determine if low-dose ^16^O radiation affected short-term spatial memory [23]. The amount of time a mouse spends exploring the novel arm of the maze compared to the familiar arm in the testing phase is indicative of its ability to retain the spatial memory encoded during the familiarization session. We observed that all irradiated groups displayed significant differences in exploration between the maze arms during the testing phase (0 Gy: F_(2,25)_ = 42.49, *p* < 0.0001, Figure 1a; 0.05 Gy: F_(2,26)_ = 29.54, *p* < 0.0001, Figure 1b). We then calculated the discrimination ratios for the Y-maze to help characterize each animal’s ability to remember a novel arm or object. All cohorts showed positive discrimination ratios, and there was no effect of radiation on discrimination ratios (*t* = 0.6801, *p* = 0.51; Figure 1c).

**Figure 2:**
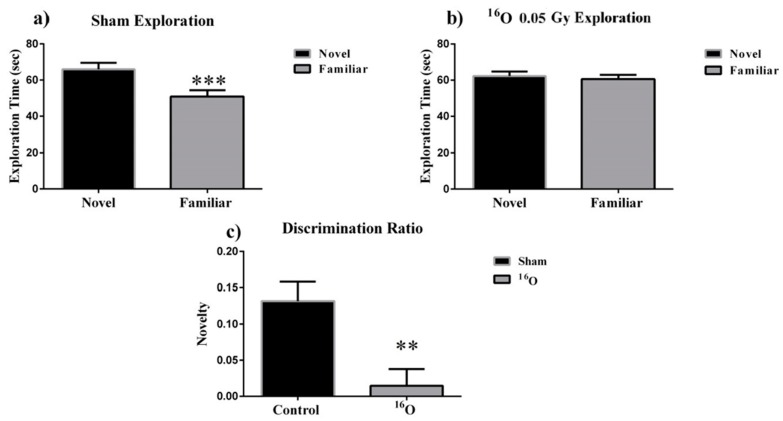
Novel object recognition. (**a**) sham-irradiated animals spent significantly more time exploring the novel object. (**b**) Irradiated groups were unable to discern the novel from the familiar object on test day, whereas (**c**) radiation induced decreased discrimination ratios, representing an inability to discriminate between the novel and familiar objects. Average ± SEM (*n* = 12); ** *p* < 0.01, *** *p* < 0.001.

Next, we used the NOR task to determine if low-dose ^16^O radiation affected non-spatial declarative memory [24]. Rodents naturally orient their head toward novel stimuli, behavior that provides a simple and effective method for quantifying recognition. Rodents display neophilia with increased stimulus exposure and contrasting exploration of a novel versus a familiar object provides an index of object recognition and discrimination [25]. Visuospatial orientation toward an object decreases with exposure time (habituation), and contrasting exploration of a novel versus familiar object provides an index of object recognition and discrimination. During familiarization (day 3), mice were placed in the open field box with 2 identical objects. On day 4, one of the objects (henceforth “familiar”) was replaced with a novel object. We found that exposure to 0.05 Gy ^16^O significantly impaired mice such that they did not show any preference for the novel object over the familiar object (*t* = 0.5823, *p* = 0.57, Figure 2b). Radiation also resulted in a significant decreased discrimination ratio for mice irradiated with 0.05 Gy (*t* = 3.800, *p* < 0.01, Figure 2c). In contrast, sham-irradiated mice showed novel object recognition and visited the novel object more often than the familiar one (*t* = 5.357, *p* < 0.001; Figure 2a).

### 2.2. Dendritic Morphology

**Figure 3:**
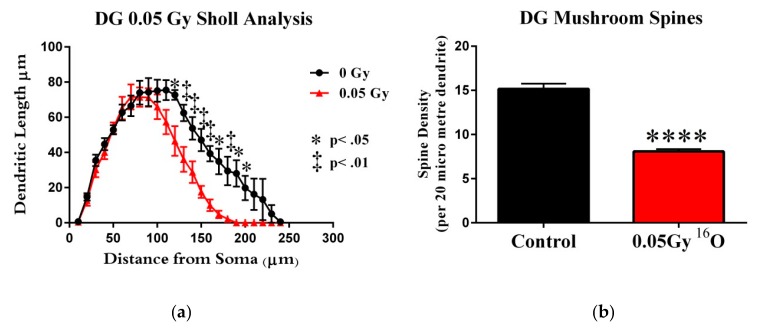
Dentate gyrus (DG) dendrite and spine morphology. (**a**) Dendritic length reduces significantly due to 0.05 Gy of ^16^O. (**b**) Irradiation significantly reduced mushroom spine density in the DG. Average ± SEM (*n* = 5); **** *p* < 0.0001.

*Dentate gyrus granule neurons*. We performed Sholl analyses to investigate the effects of ^16^O on dendritic length (assaying the distance from the soma in 10-µm intervals). We detected significant interactions between treatment and dendritic length (F_(23,184)_ = 2.78; *p* < 0.0001), indicating that the effect of radiation was associated with a different distribution of dendritic branches over the entire tree. We also found significant main effects on Sholl dendritic length after 0.05 Gy irradiation (F_(23,184)_ = 50.00; *p* < 0.0001) and treatment (F_(1,8)_ = 35.77; *p* < 0.001). Post-hoc analysis revealed that irradiation decreased dendritic arborization compared to the sham controls. We observed significant decreases in the dendritic arbor at a distance of 120–190 µm from the soma after 0.05 Gy (Holm-Sidak 120–140 µm, *p* < 0.05; 150–190 µm, *p* < 0.01; Figure 3a). In addition, we observed significant decreases in the number of branch points (*t* = 3.20, *p* < 0.05), dendritic length (*t* = 4.84, *p* < 0.01), tips (*t* = 2.83, *p* < 0.05; Table 1), and dendritic complexity (*t* = 3.81, *p* < 0.01; Table 1) in irradiated animals compared to controls. These data indicate that ^16^O irradiation decreased dendritic complexity in the DG region of the hippocampus.

**Figure 4:**
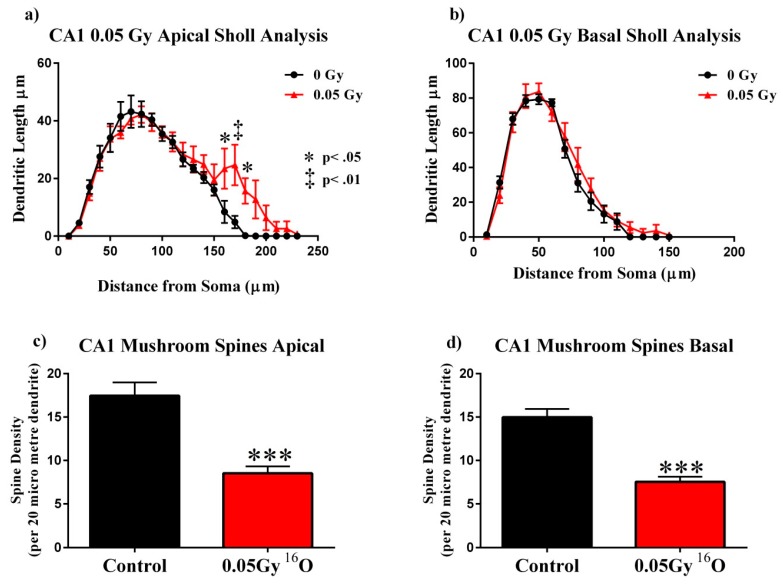
Cornu Ammonis 1 (CA1) dendritic morphology. (**a**) The apical CA1 underwent significant increase in dendritic length beginning at approximately 150 µm from the soma. (**b**) Radiation dosage did not affect morphology in Basal CA1. (**c**,**d**) Radiation significantly decreased mushroom spine morphology in CA1 Apical and Basal. Average ± SEM (*n* = 5); *** *p* < 0.001.

Cornu Ammonis 1 (CA)1 pyramidal neurons. Next, we analyzed differences in the CA1 neurons between groups. We found significant interactions between treatment and dendritic Sholl length in the apical subregion (0.05 Gy: F_(22,176)_ = 2.144; *p* < 0.05). However, we did not see significant interactions between treatment groups and dendritic Sholl length in the basal subregion. We also found significant main effects on Sholl dendritic length after 0.05 Gy radiation in the apical (F_(22,176)_ = 45.97; *p* < 0.0001) and basal (F_(14,112)_ = 110.1; *p* < 0.0001) subregions. Post-hoc multiple comparisons revealed significant increases in dendritic length after 0.05 Gy irradiation at 160–180 µm (Holm-Sidak 160 and 180 µm, *p* < 0.05; 170 µm, *p* < 0.01; Figure 4a) from the soma in the apical CA1. However, we saw no significant increases in dendritic length in the basal CA1 (Figure 4b).

In contrast to the observation in the DG, no differences were found in the CA1 apical and basal area for total dendritic length (Apical *t* = 0.82, *p* = 0.43; Basal *t* = 0.85, *p* = 0.42), the number of branch points (Apical *t* = 0.34, *p* = 0.74; Basal *t* = 1.53, *p* = 0.16), branch tips (Apical *t* = 0.25, *p* = 0.80; Basal *t* = 1.34, *p* = 0.21; see Table 2), or the branch point complexity (Apical *t* = 0.64, *p* = 0.54; Basal *t* = 0.14, *p* = 0.88).

**Figure 5:**
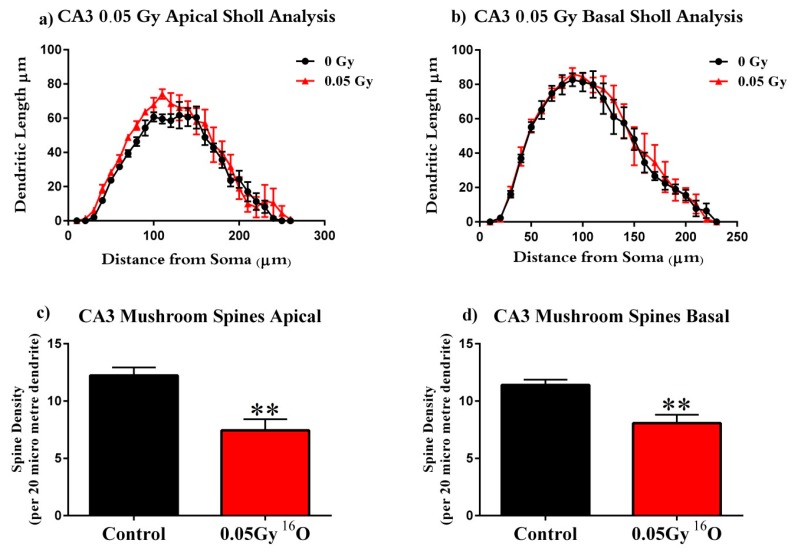
CA3 Sholl analyses and spine morphology. (**a**,**b**) 0.05 Gy radiation did not affect arborization CA3 apical and basal pyramidal dendrites. (**c**,**d**) Mushroom spine density was significantly lowered as a result of treatment. Average ± SEM (*n* = 5); ** *p* < 0.01.

*CA3 pyramidal neurons*. Finally, we conducted Sholl analyses in the hippocampal CA3. In contrast to what was observed in CA1 spine analysis, there was no significant interaction between radiation and segmental dendritic length in the CA3 apical area (F_(25,200)_ = 0.86; *p* = 0.66; Figure 5a) or in the basal dendrites (F_(22,176)_ = 0.24; *p* = 0.99). Similar to the observation in the CA 1, no differences were found in the CA3 apical and basal area for total dendritic length (Apical *t* = 0.73, *p* = 0.48; Basal *t* = 0.41, *p* = 0.42), the number of branch points (Apical *t* = 1.21, *p* = 0.25; Basal *t* = 2.03, *p* = 0.07), branch tips (Apical *t* = 2.04, *p* = 0.07; Basal *t* = 1.67, *p* = 0.13; see Table 3), or the branch point complexity (Apical *t* = 0.10, *p* = 0.91; Basal *t* = 1.60, *p* = 0.14).

### 2.3. Spine Morphology

*Dentate gyrus granule neurons*. Our quantitative analyses showed that ^16^O radiation did not affect overall spine density in the DG (*t* = 1.544, *p* = 0.13; Table 1). Next, we analyzed the density of specific types of dendritic spines. We found that stubby spine density (*t* = 0.6896, *p* = 0.49; Table 1) was not altered by radiation. However, we observed a decrease in mushroom spine density (*t* = 10.86, *p* < 0.0001; Figure 3b) and an increase in thin spine density (*t* = 2.260, *p* = 0.03; Table 1) after irradiation relative to controls.

*CA1 pyramidal neurons*. As observed for overall DG spine density, we did not observe radiation-induced changes to overall CA1 apical spine density (apical: *t* = 0.7133, *p* = 0.49; Table 2a). When we analyzed the density by spine type, we found a non-significant decrease in stubby spine density after irradiation (*t* = 0.4049, *p* = 0.69; Table 2a). However, we found a significant increase in thin spine density (*t* = 2.608, *p* = 0.03; Table 2a) and a significant decrease in mushroom spine density (*t* = 5.208, *p* = 0.0008; Figure 4a).

In CA1 basal dendrites, we also found no significant radiation-induced changes in overall spine density compared to controls (*t* = 0.9543, *p* = 0.36; Table 2b). Likewise, there were no significant changes in stubby spine density (*t* = 0.2665, *p* = 0.79; Table 2b). However, there were significant increases in thin spine density (*t* = 3.706, *p* = 0.006; Table 2b) and significant decreases in mushroom spine density after irradiation (*t* = 6.668, *p* = 0.0002; Figure 4d).

*CA3 pyramidal neurons*. Irradiation did not significantly alter the overall density of CA3 apical spines (apical: *t* = 0.3508, *p* = 0.73; Table 3a). When considering individual spine types, we found a non-significant increase in thin spine density (*t* = 1.708, *p* = 0.13; Table 3a) and a non-significant decrease in stubby spine density (*t* = 0.4377, *p* = 0.67; Table 3a). However, we observed a significant decrease in mushroom spine density (*t* = 4.083, *p* = 0.004; Figure 5c). In the CA3 basal spines, irradiation did not significantly alter the overall spine density compared to controls (*t* = 0.2783, *p* = 0.78; Table 3b). In addition, we did not observe a significant change in thin spine density (*t* = 1.062, *p* = 0.32; Table 3b) and stubby spine density (*t* = 0.2646, *p* = 0.79; Table 3b). However, there were significant decrease mushroom spine density (*t* = 3.98, *p* = 0.005; Figure 5d).

### 2.4. NMDA/AMPA Subunits

**Figure 6:**
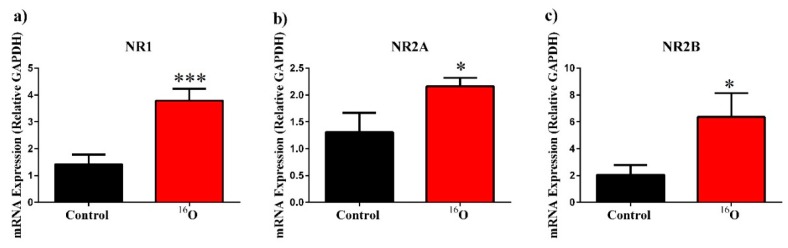
Alterations in *N*-methyl-d-aspartate (NMDA) mRNA expression. ^16^O significantly up-regulates expression of (**a**–**c**) NMDA receptor subunits NR1, NR2A and NR2B. Average ± SEM (*n* = 10)); * *p* < 0.05, *** *p* < 0.001.

We examined the mRNA expression of glutamate receptors in response to radiation. NMDA subunits Nr1 (*t* = 4.18, *p* = 0.0007; Figure 6a), Nr2a (*t* = 2.50, *p* = 0.02: Figure 6b) and Nr2b (*t* = 2.14, *p* = 0.04: Figure 6c) underwent a significant increase in expression.

### 2.5. Synaptic Markers

**Figure 7:**
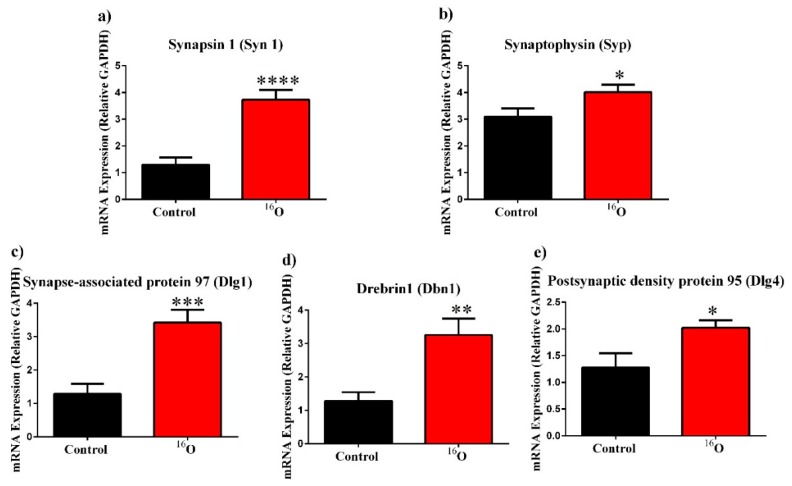
Alterations in synaptic marker mRNA expression. ^16^O significantly up-regulates expression of (**a**,**b**) Presynaptic vesicle trafficking marker Synapsin 1 and marker of synaptic density Synaptophysin. (**c**–**e**) Postsynaptic density markers Synapse associated protein 97, Drebrin 1 and Postsynaptic density protein 95 were up-regulated by radiation Average ± SEM (*n* = 10); * *p* < 0.05, ** *p* < 0.01, *** *p* < 0.001, **** *p* < 0.0001.

We analyzed the mRNA expression of presynaptic markers of synapsin-1 (*t* = 4.30, *p* = 0.0001; Figure 7a) and Synaptophysin (*t* = 2.50, *p* = 0.03: Figure 7b) underwent a significant increase in expression. Radiation exposure to postsynaptic markers Synapse-Associated Protein 97 (*t* = 4.34, *p* = 0.0005: Figure 7c), Drebrin 1 (*t* = 3.24, *p* = 0.003: Figure 7d) and Postsynaptic density protein 95 (*t* = 2.54, *p* = 0.02: Figure 7e) also resulted in significant increases in mRNA expression.

## 3. Discussion

Recognition memory is defined as the ability to identify a previously encountered object and/or situation as familiar [26]. In this study, we found that 0.05 Gy of ^16^O-particle radiation significantly impaired recognition memory up to 9 months after irradiation. In agreement with our results, previous studies reported a deficit in NOR 2 months after exposure to 0.05 Gy of ^16^O radiation [16]. Likewise, deficits in NOR (increased exploration of the familiar object) were also noted 6 weeks after irradiation with the same dosage and isotope [27]. Mice exposed to ^16^O (0.3 Gy) and ^48^Ti (0.05 or 0.3 Gy) radiation failed to discriminate between novel and familiar objects during the novel object recognition test and had a reduced preference to explore the novelty during the object in place tasks [15]. Likewise, rats irradiated with 0.5 Gy of ^16^O spent significantly less time exploring the novel object in the novel object recognition paradigm [16]. In the past, we observed detrimental effects of 0.1 and 0.25 Gy of ^16^O radiation on short-term spatial memory during Y-maze testing. However, 0.05 Gy dose of radiation did not seriously affect short-term spatial memory. This suggests that radiation dose impacts the extent to which short-term spatial memory is altered in mice.

There are three morphological classes of dendritic spines, with the name of each class based on their size and shape: (1) Mushroom spines, which have complex postsynaptic densities with a more glutamate receptors than other spines [28]; (2) stubby spines, which lack a neck; and (3) thin spines, which contain a small bulbous head and a long, thin neck [29]. Data from us and others show that high-LET doses from 0.1–1 Gy cause significant, dose-responsive reductions in hippocampal dendritic complexity and spine density, which last at least one month post-irradiation [21,22,30].

In the current study, 9 months post irradiation we also observed significant decreases in mushroom spine density in the DG, CA1, and CA3 within the hippocampus, and there was a significant increase in thin spine density in two of these three regions. There is a strong correlation between the size of the dendritic spine head and the strength of the synapse [31], and abnormalities in spine number and morphology have been observed in a number of neurological disorders [32]. Mushroom spines are known as “memory spines”, due to their stability and postsynaptic operation [33,34]. The decrease in DG, CA1 and CA3 mushroom spines is consistent with the observed behavior of irradiated mice who demonstrated diminished memory and recall during the NOR test.

Dendrites are the main portion of neurons that receive and process presynaptic input [35]. They have a complex geometry involving processes where the proximal branches have a larger diameter than the distal branches. As dendrites develop, they form several connections with other neurons in a process referred to as “dendritic arborization”. The extent and pattern of this branching determines the amount of synaptic inputs a dendrite can adequately process. Previously, our group investigated the effects of whole-body irradiation with 0.1, 0.25, or 1 Gy of ^16^O on dendritic morphology 2 weeks post irradiation. All doses of ^16^O radiation decreased dendritic branch points (indicated by bifurcation), dendritic length, and complexity [18]. In agreement with our previous study, 0.05 Gy ^16^O significantly decreased dendritic length and complexity in the DG. This is also in agreement with a previous study where reductions in the DG dendritic structure were observed after 0.1 and 1 Gy whole-body ^1^H irradiation [36]. In that study, significant changes in the number of dendritic branches, branch points, and dendritic length were observed 30 days after irradiation, indicating a reduction in dendritic complexity.

The cellular foundation of learning and memory involves synaptic plasticity which comprises strengthening the effectiveness of synapses [29,37]. The enlargement of dendritic spines and formation of new spines is dependent upon the NMDA receptor [38,39]. In the DG and CA 1 apical and basal, the density of thin spine was significantly increased. Thin spines are thought to be more plastic linked to learning as containing a predominantly greater number of the NMDA glutamate receptors. This increase in thin spines may contribute to the increase in NMDA mRNA [40]. Functional NR1/NR2A receptors have a particularly close relationship with long-term potentiation (LTP) that is a form of synaptic plasticity [41]. Lack of, or hypoactivation of NMDA receptors is linked to cognitive deficits and has been implicated in schizophrenia and Alzheimer’s disease [42]. Cranial γ-irradiation has been previously shown to increase NMDA receptor subunits NR1 and NR2A in the CA1 of rats [43]. In the current study, we observed a significant increase in NR1, NR2A and NR2B expression in the hippocampus after ^16^O exposure, which is consistent with these findings. NMDARs (*N*-methyl-d-aspartate (NMDA) receptors) are important for cell survival but can also be harmful and kill neurons. Activation of synaptic NMDAR is suggested to improve synaptic plasticity and learning and memory ability, and promotes neuronal survive and maturation, while activation of extrasynaptic NMDAR could induce neuronal death, synaptic plasticity failure and memory loss. These extrasynaptic NMDARs comprise both NR2A- and NR2B-containing receptors [44]. Stimulation of synaptic NMDARs induces prosurvival events through the activation of cAMP response element-binding protein and the extracellular signal-regulated kinase [45]. Conversely, calcium flux through extrasynaptic NMDARs overrides these functions, causing mitochondrial dysfunction and cell death. It is possible that sustained large increases in intracellular calcium through glutamate receptor channels represent a final common pathway of neuronal cell death that is associated with neurodegenerative diseases [41]. In addition, mice overexpressing NR2A exhibit impairment in long-term memory, but not in short-term memory tests, suggesting that the consolidation process is compromised [36]. Consequently, in the current study we did not see a deficit in short term memory tested in the Y-maze (Figure 1). However, a deficit in consolidation was detected during the novel object recognition paradigm (Figure 2).

In the current study, we observed significant upregulation of Synapsin 1 (Syn 1), Synaptophysin (Syp), Synapse-associated protein (SAP97), and Drebrin 1 (Dbn1) Post-synaptic density protein (PSD-95) mRNA expression in the whole hippocampus 9 month after 0.05 Gy ^16^O irradiation exposure. Syn 1 is a member of a family of terminal specific phosphoproteins involved in synaptic vesicle clustering and release, which mediates synaptic transmission. Syp a glycosylated polypeptide located in the synaptic vesicle membrane plays an important role in regulating neurotransmitter release [46,47]. PSD-95 and SAP97 are post-synaptic density molecules, and their presence is crucial for normal electrical activity [48,49]. Increases in PSD-95 expression have been previously observed as a result of ^16^O irradiation (0.05 and 0.3 Gy, 6-weeks post exposure) in the medial prefrontal cortex [15]. In addition, an increase in PSD-95 expression has also been observed after ^1^H irradiation (0.1 and 1 Gy, 30 days post exposure) in the DG [50]. These data indicate that ^16^O irradiation induces high expression of synaptic function/structure genes that may have profound effects on the overall stability of synaptic connections in the hippocampus leading to hyperactivity. In conclusion, this study provides evidence that long term exposure to low dose ^16^O radiation has the ability to impair cognition, compromise the integrity of dendrite morphology in the hippocampus, and impact the expression of glutamate receptors that are involved in learning and memory.

## 4. Materials and Methods

### 4.1. Animals and Irradiation

The work was performed under animal use protocol 3523 approved by the University of Arkansas for Medical Sciences (UAMS) Institutional Animal Care and Use Committee on 24 June 2014.

Male C57BL/6 mice were purchased from The Jackson Laboratory (Bar Harbor, ME, USA) at 2 months old. Five animals were housed in each cage; they received standard low-soy rodent chow (2020X; Harlan^®^ Laboratories Inc., Indianapolis, IN, USA) and water ad libitum in 12:12 hour light–dark housing at UAMS. All cages were kept in the same ventilated cabinet in the animal facility. Once the animals were 6 months old, they were airlifted overnight to Brookhaven National Laboratory (BNL), where they lived under the same housing conditions. After acclimating for 1 week, they were exposed to whole-body ^16^O-particle radiation at doses of 0 and 0.05 Gy (600 MeV/n, 18–33 Gy/min) at the NASA Space Radiation Laboratory (NSRL) at BNL. For irradiation, mice were placed in well-ventilated acrylic cubes and situated on foam holders before being placed on the beam line. Sham-irradiated animals were also placed on the beam line. Dosimetry was carried out by NSRL physicists. Two days after irradiation, animals were airlifted back to UAMS and underwent a standard 8-week quarantine protocol where they were fed 2020X chow containing 150-ppm fenbendazole. Seven days prior to testing, animals were subjected to a handling protocol, where the technician placed their hand in the cage allowing the mice to sniff and explore at will for 5 min. Cages were removed in pairs from their racks and placed on carts for the handling. Mice were transported to the testing room in their home cages, at least 1 h prior to the start of behavioral testing. Individual mice can then be transported singly, in clean cages, into the testing apparatus. All animal procedures were approved by the Institutional Animal Care and Use Committees of UAMS and BNL.

### 4.2. Y-Maze

**Scheme 1:**
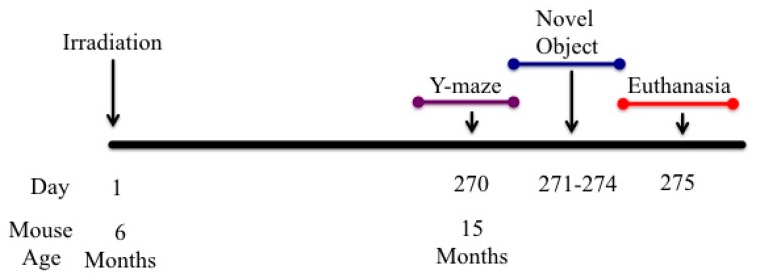
Schematic diagram showing experimental design. Six-month-old C57BL/6J male mice received whole-body irradiation ^16^O radiation at doses of 0, 0.05 Gy (600 MeV/n) at the National Aeronautics and Space Administration (NASA)’s Space Radiation Laboratory (NSRL) within Brookhaven National Laboratory (BNL); 270 days post irradiation behavioral testing was performed.

Animal behavior was tested 9 months after irradiation (*n* = 12 per treatment) (Scheme 1). Mice were first tested in the Y-maze, which does not rely on either negative or positive reinforcement. The maze is constructed out of acrylic and consists of 3 similar arms (45 L × 7 W × 14 H cm): a “start” arm where animals are placed initially, a “familiar” arm, and a “novel” arm. The familiar and novel arms each contained an object of different size and shape mounted at the end of the arm. Animals were placed in the start arm facing away from the center of the maze. The familiarization session consisted of free exploration of the start and familiar arms for 10 min. Four hours later, the testing session was held; animals were again placed in the maze, this time with access to all arms. Allocation of arms (start, familiar, or novel) was counterbalanced between each experimental group. Trials lasted for 10 min, and center and nose-points were recorded throughout each session. All experimental arenas were wiped clean with 20% ethanol after each trial. All behavioral experiments were conducted during the light cycle under dimly-lit (white light) conditions, after a minimum of one hour of acclimation. Behavioral experiments were recorded on a charge-coupled device video camera, located above the maze for automatic behavioral analysis with EthoVision^®^ software version 11 (Noldus Information Technology, Leesburg, VA, USA).

### 4.3. Novel Object Recognition

Animals were tested for NOR with a 4-day procedure in which animals freely explored an arena for 10 min each day. The arena is a cube consisting of an aluminum floor, acrylic walls (41 × 41 × 35 cm), and an open ceiling. The first 2 days served as habituation learning days, in which mice were able to explore the empty arena (effectively serving as open field tests); locomotor activity was measured at this stage. The familiarization phase occurred on day 3, when animals explored an arena containing 2 identical objects (cell-culture flasks filled with sand). Novel object recognition testing occurred on day 4; here, a now-familiar object was replaced with a novel object (large LEGO^®^ blocks assembled to the size of the cell-culture flasks) [51]. Animals were placed in the center of the arena parallel to the objects to avoid bias. NOR testing relies on the animals’ natural inclination to explore novel objects in their environment (untreated animals should spend significantly more time exploring the novel object). The tracking software was programmed to track animal center-points for the habituation trials and nose-points during familiarization and testing trials.

### 4.4. RNA Extraction and Quantitative Reverse Transcription Polymerase Chain Reaction (qRT-PCR)

The day after behavioral testing, animals were anesthetized with isoflurane, their brains were subsequently collected and dissected along the midsagittal plane (*n* = 5–6), immediately frozen in liquid nitrogen, and subsequently stored at −80 °C. Total RNA was extracted from hippocampal tissue with the AllPrep DNA/RNA extraction kit (QIAGEN, Valencia, CA, USA), according to the manufacturer’s protocol. RNA quality and quantity were assessed on a Nanodrop 2000 instrument (Thermo Fisher Scientific, Waltham, MA, USA). cDNA was synthesized with random primers and a high-capacity cDNA reverse transcription kit (Applied Biosystems, Foster City, CA, USA), according to the manufacturer’s protocol (Life Technologies, Grand Island, NY, USA). The levels of gene transcripts were determined by qRT-PCR with TaqMan Gene Expression Assays (Life Technologies, and Integrated DNA Technologies, Coralville, IA, USA), according to the manufacturer’s protocol. In all cases, Glyceraldehyde 3-phosphate dehydrogenase GAPDH was used as an internal reference gene, and fold changes were calculated with the 2^−dd*C*t^ method. Measurements were taken in duplicates (Table 4).

### 4.5. Golgi Staining

We adapted a Golgi-staining protocol using reagents from the superGolgi Kit (Bioenno Tech, Santa Ana, CA, USA) [52]; Golgi-staining is a reliable method for assessing dendrites and dendritic spine dynamics [30,53]. Right hemispheres were immediately impregnated in a potassium dichromate solution for 2 weeks (*n* = 6). Next, sections were immersed for at least 48 h in a post-impregnation buffer. Samples were sectioned at 200 µm in 1× PBS along the coronal plane; they were then transferred into wells and washed with 0.01 M PBS-T buffer (pH 7.4, 0.3% Triton X-100). Immediately after washing, samples were stained with ammonium hydroxide and then immersed in a post-staining buffer. Sections were again washed with PBS-T, mounted on 1% gelatin-coated slides, and allowed to dry. The sections were then dehydrated with ethanol solutions, cleaned with xylene, and cover-slipped with Permount^TM^ (Thermo Fisher Scientific, Waltham, MA, USA).

### 4.6. Dendritic Morphology

Researchers were blinded to the experimental conditions when collecting dendritic morphology data. We quantified the morphological characteristics of granular and pyramidal neurons in the hippocampus via Sholl analyses, total dendritic length, number of branch points, and the dendritic complexity index (DCI) using the Neuroexplorer component of the Neurolucida program (Ver. 11, Microbrightfield, Inc., Williston, VT, USA). Sholl analysis is used to assess the amount and distribution of the arbor at increasing radial distances from the cell body [54]. Radii were set to extend in 10-µm intervals from the soma. The length of each dendritic branch, within each progressively larger circle, was counted from the soma, with respect to 3 dimensions.

We then performed branch-point analyses to determine the complexity of dendritic arborization; the complexity of the dendritic tree is an important phenotypic component of branching analysis. The DCI was determined with the equation DCI = ∑ (branch tip orders + # branch tips) × (total dendritic length/total number of primary dendrites). Apical and basal dendrites were analyzed separately in the cornu Ammonis (CA)-1 and CA3 areas. We traced 5 randomly stained neurons per subregion per animal.

### 4.7. Dendritic Spine Density and Morphology

We analyzed dendritic spines in coded Golgi-impregnated brain sections from the dorsal and ventral hippocampus. Spines were examined on dendrites of dorsal dentate gyrus (DG) granule neurons and on apical (stratum radiatum) and basal (stratum oriens) dendrites of the dorsal CA1and CA3 pyramidal neurons. The neurons that satisfied the following criteria were chosen for analysis in each of the experimental groups: (1) presence of non-truncated dendrites; (2) consistent and dark Golgi-staining along the extent of the dendrites; and (3) relative isolation from neighboring neurons to avoid interference with analysis [55]. Five dendritic segments per neuron were analyzed (each at least 20 nm long), and 6–7 neurons were analyzed per brain [56]. Neurons that met staining criteria were traced using a 100× oil objective, a computerized stage, and Neurolucida software (Ver. 11, Microbrightfield, Inc.).

### 4.8. Statistical Analyses

We expressed data as the mean ± the standard error of the mean (SEM). Behavioral assays comparing visits or time spent in apparatus areas among individual treatment groups were analyzed with unpaired *t*-tests. Discrimination ratios (DR) were calculated with the following formulas: (*Y-Maze*) DR = (time spent exploring novel arm − time spent exploring familiar arm)/(time spent exploring novel arm + time spent exploring familiar arm); (*NOR*) DR = (novel object visits − familiar object visits)/(novel object visits + familiar object visits). We used a one-way analysis of variance (ANOVA) and a Bonferroni post-hoc test to evaluate statistical differences between sham and irradiated groups in measures of discrimination ratios, the dendritic complexity index, and spine density. Sholl analyses were conducted via a mixed-factors ANOVA to test for the effect of treatment on dendritic distance from the soma (the Sholl radius, being a repeated measures variable); Holm-Sidak multiple comparison was used when appropriate. Dendritic spine data were compared via ANOVA with post-hoc Holm-Sidak corrections for multiple comparisons. To evaluate statistical differences in *N*-methyl-d-aspartate (NMDA) and Synaptic Markers mRNA expression between sham and irradiated groups, we used the paired *t*-test. All statistical analyses were conducted with GraphPad Prism 6.0 software (La Jolla, CA, USA) in a 95% confidence interval, and *p* < 0.05 was considered significant.

## Figures and Tables

**Table 1:** Effects of ^16^O on dendrite morphology in the DG.

Cell Type and Measurement	Sham (Mean ± SEM)	0.05 Gy (Mean ± SEM)	*p*-Value
DG			
Thin Spines	58.00 ± 1.15	62.97 ± 1.87	***p = 0.03***
Stubby Spines	28.43 ± 1.98	29.98 ± 1.07	*p* = 0.49
Mushroom Spines	15.16 ± 0.59	8.078 ± 0.26	***p = 0.0001***
Overall Density	24.28 ± 0.56	22.88 ± 0.70	*p* = 0.13
Total Dendritic Length (µm)	919.3 ± 38.51	664.8 ± 35.68	***p = 0.001***
Total # Branch Points	6.160 ± 0.34	4.880 ± 0.20	***p = 0.01***
Total # Branch Tips	7.840 ± 0.35	6.640 ± 0.23	***p = 0.02***
Dendritic Complexity	17528 ± 1328	10307 ± 1351	***p = 0.005***

**Table 2:** Effects of ^16^O on dendrite morphology in the CA1.

**(a)**			
**Cell Type and Measurement**	**Sham (Mean ± SEM)**	**0.05 Gy (Mean ± SEM)**	***p*-Value**
**CA1 Apical**			
Thin Spines	53.85 ± 3.09	63.97 ± 2.33	***p = 0.03***
Stubby Spines	28.69 ± 1.90	27.51 ± 2.19	*p* = 0.69
Mushroom Spines	17.47 ± 1.51	8.522 ± 0.80	***p = 0.0008***
Overall Density	22.54 ± 0.82	19.33 ± 1.11	*p* = 0.49
Total Dendritic Length (µm)	382.6 ± 27.74	414.6 ± 27.74	*p* = 0.43
Total # Branch Points	4.04 ± 0.39	4.20 ± 0.25	*p* = 0.74
Total # Branch Tips	5.08 ± 0.40	5.20 ± 0.25	*p* = 0.81
Dendritic Complexity	7934 ± 1499	9121 ± 1058	*p* = 0.53
**(b)**			
**Cell Type and Measurement**	**Sham (Mean ± SEM)**	**0.05 Gy (Mean ± SEM)**	***p*-Value**
**CA1 Basal**			
Thin Spines	54.99 ± 1.53	62.94 ± 1.49	***p = 0.006***
Stubby Spines	30.03 ± 0.65	29.51 ± 1.82	*p* = 0.7966
Mushroom Spines	14.98 ± 0.95	7.54 ± 0.57	***p = 0.0002***
Overall Density	21.75 ± 0.73	22.61 ± 0.52	*p* = 0.36
Total Dendritic Length (µm)	441.5 ± 10.15	467.7 ± 29.29	*p* = 0.42
Total # Branch Points	3.16 ± 0.37	3.92± 0.32	*p* = 0.16
Total # Branch Tips	6.48 ± 0.25	7.07 ± 0.35	*p* = 0.21
Dendritic Complexity	2715 ± 859.4	2843 ± 251.8	*p* = 0.88

**Table 3:** Effects of ^16^O on dendrite morphology in the CA3.

**(a)**			
**Cell Type and Measurement**	**Sham (Mean ± SEM)**	**0.05 Gy (Mean ± SEM)**	***p*-Value**
**CA 3 Apical**			
Thin Spines	56.46 ± 2.76	62.45 ± 1.80	*p* = 0.13
Stubby Spines	31.36 ± 2.22	30.11 ± 1.53	*p* = 0.67
Mushroom Spines	12.23 ± 0.70	7.44 ± 0.97	***p = 0.004***
Overall Density	22.92 ± 0.83	23.38 ± 1.00	*p* = 0.73
Total Dendritic Length (µm)	715.0 ± 50.22	794.2 ± 96.01	*p* = 0.48
Total # Branch Points	5.240 ± 0.27	5.720 ± 0.28	*p* = 0.25
Total # Branch Tips	6.680 ± 0.33	7.720 ± 0.38	*p* = 0.07
Dendritic Complexity	14009 ± 1362	14239 ± 1742	*p* = 0.91
**(b)**			
**Cell Type and Measurement**	**Sham (Mean ± SEM)**	**0.05 Gy (Mean ± SEM)**	***p*-Value**
**CA3 Basal**			
Thin Spines	57.23 ± 2.01	59.79 ± 0.86	*p* = 0.32
Stubby Spines	31.49 ± 1.90	32.14 ± 1.33	*p* = 0.79
Mushroom Spines	11.40 ± 0.46	8.066 ± 0.74	*p* = 0.28
Overall Density	22.90 ± 0.22	22.51 ± 1.57	*p* = 0.78
Total Dendritic Length (µm)	863.5 ± 88.53	912.2 ± 78.00	*p* = 0.69
Total # Branch Points	5.760 ± 0.56	6.960 ± 0.17	*p* = 0.07
Total # Branch Tips	8.200 ± 0.61	9.360 ± 0.31	*p* = 0.13
Dendritic Complexity	11592 ± 1926	16327 ± 2240	*p* = 0.14

**Table 4:** TaqMan Assay IDs for real-time polymerase chain reaction (PCR).

Assay ID	Gene Symbol
Mm00433790_m1	NR1: mCG20293
Mm00433802_m1	NR2A: mCG123867
Mm00433820_m1	NR2B: mCG145739
Mm00436850_m1	Syp: mCG3961
Mm01344468_m1	Dlg1: mCG129938
Mm00517314_m1	Dbn1: mCG3204
Mm00492193_m1	Dlg4: mCG22507
Mm00449772_m1	Syn1: mCG117699

## References

[1] Sarafin M. (2017). NASA|Exploration Mission-1—Pushing Farther into Deep Space.

[2] Schimmerling W. Acceptablerisk. https://three.jsc.nasa.gov/articles/AcceptableRiskWS.pdf.

[3] (2014). Human Exploration of Mars Design Reference Architecture 5.0 Addendum #2.

[4] National Research Council (U.S.). Committee for Evaluation of Space Radiation Cancer Risk Model, National Research Council (U.S.). Space Studies Board (2012). Technical Evaluation of the NASA Model for Cancer Risk to Astronauts Due to Space Radiation.

[5] Charles W., Lloyd S.T. Space Radiation. https://www.nasa.gove/sites/default/files/atoms/files/nasa_space_radiation_ebook_0.pdf.

[6] Townsend L.W. (2005). Implications of the space radiation environment for human exploration in deep space. Radiat Prot Dosim..

[7] Hellweg C.E., Baumstark-Khan C. (2007). Getting ready for the manned mission to mars: The astronauts’ risk from space radiation. Die Naturwissenschaften.

[8] Fike J.R., Gobbel G.T., Gutin P.H., Leibel S.A., Sheline G.E. (1991). Central nervous system radiation injury in large animal models. Radiation Injury to the Nervous System.

[9] Tofilon P.J., Fike J.R. (2000). The radioresponse of the central nervous system: A dynamic process. Radiat. Res..

[10] Abayomi O.K. (1996). Pathogenesis of irradiation-induced cognitive dysfunction. Acta Oncol..

[11] Shukitt-Hale B., Casadesus G., McEwen J.J., Rabin B.M., Joseph J.A. (2000). Spatial learning and memory deficits induced by exposure to iron-56-particle radiation. Radiat. Res..

[12] Shukitt-Hale B., Casadesus G., Cantuti-Castelvetri I., Rabin B.M., Joseph J.A. (2003). Cognitive deficits induced by 56fe radiation exposure. Adv. Space Res..

[13] Manda K., Ueno M., Anzai K. (2008). Space radiation-induced inhibition of neurogenesis in the hippocampal dentate gyrus and memory impairment in mice: Ameliorative potential of the melatonin metabolite, afmk. J. Pineal Res..

[14] Rabin B.M., Carrihill-Knoll K.L., Miller M.G., Shukitt-Hale B. (2018). Age as a factor in the responsiveness of the organism to the disruption of cognitive performance by exposure to HZE particles differing in linear energy transfer. Life Sci. Space Res..

[15] Parihar V.K., Allen B., Tran K.K., Macaraeg T.G., Chu E.M., Kwok S.F., Chmielewski N.N., Craver B.M., Baulch J.E., Acharya M.M. (2015). What happens to your brain on the way to mars. Sci. Adv..

[16] Rabin B.M., Poulose S.M., Carrihill-Knoll K.L., Ramirez F., Bielinski D.F., Heroux N., Shukitt-Hale B. (2015). Acute effects of exposure to ^56^Fe and ^16^O particles on learning and memory. Radiat. Res..

[17] Rabin B.M., Shukitt-Hale B., Carrihill-Knoll K.L., Gomes S.M. (2014). Comparison of the effects of partial- or whole-body exposures to ^16^O particles on cognitive performance in rats. Radiat. Res..

[18] Carr H., Alexander T.C., Groves T., Kiffer F., Wang J., Price E., Boerma M., Allen A.R. (2018). Early effects of ^16^O radiation on neuronal morphology and cognition in a murine model. Life Sci. Space Res..

[19] Gehrke A.K., Baisley M.C., Sonck A.L., Wronski S.L., Feuerstein M. (2013). Neurocognitive deficits following primary brain tumor treatment: Systematic review of a decade of comparative studies. J. Neuro-Oncol..

[20] Rao A.A., Ye H., Decker P.A., Howe C.L., Wetmore C. (2011). Therapeutic doses of cranial irradiation induce hippocampus-dependent cognitive deficits in young mice. J. Neuro-Oncol..

[21] Chakraborti A., Allen A., Allen B., Rosi S., Fike J.R. (2012). Cranial irradiation alters dendritic spine density and morphology in the hippocampus. PLoS ONE.

[22] Parihar V.K., Limoli C.L. (2013). Cranial irradiation compromises neuronal architecture in the hippocampus. Proc. Natl. Acad. Sci. USA.

[23] Dellu F., Fauchey V., Le Moal M., Simon H. (1997). Extension of a new two-trial memory task in the rat: Influence of environmental context on recognition processes. Neurobiol. Learn. Mem..

[24] Ran Y., Yan B., Li Z., Ding Y., Shi Y., Le G. (2016). Dityrosine administration induces novel object recognition deficits in young adulthood mice. Physiol. Behav..

[25] Cohen S.J., Stackman R.W. (2015). Assessing rodent hippocampal involvement in the novel object recognition task. A review. Behav. Brain Res..

[26] Squire L.R., Wixted J.T., Clark R.E. (2007). Recognition memory and the medial temporal lobe: A new perspective. Nat. Rev. Neurosci..

[27] Gregory Nelson L.S., Janice L.H. (2016). Risk of Acute and Late Central Nervous System Effects from Radiation Exposure.

[28] Bourne J.N., Harris K.M. (2008). Balancing structure and function at hippocampal dendritic spines. Annu. Rev. Neurosci..

[29] Lai K.O., Ip N.Y. (2013). Structural plasticity of dendritic spines: The underlying mechanisms and its dysregulation in brain disorders. Biochim. Biophys. Acta.

[30] Zhao Z.H., Zheng G., Wang T., Du K.J., Han X., Luo W.J., Shen X.F., Chen J.Y. (2018). Low-level gestational lead exposure alters dendritic spine plasticity in the hippocampus and reduces learning and memory in rats. Sci. Rep..

[31] Yuste R., Bonhoeffer T. (2001). Morphological changes in dendritic spines associated with long-term synaptic plasticity. Annu. Rev. Neurosci..

[32] Van Spronsen M., Hoogenraad C.C. (2010). Synapse pathology in psychiatric and neurologic disease. Curr. Neurol. Neurosci. Rep..

[33] Trachtenberg J.T., Chen B.E., Knott G.W., Feng G., Sanes J.R., Welker E., Svoboda K. (2002). Long-term in vivo imaging of experience-dependent synaptic plasticity in adult cortex. Nature.

[34] Kasai H., Matsuzaki M., Noguchi J., Yasumatsu N., Nakahara H. (2003). Structure-stability-function relationships of dendritic spines. Trends Neurosci..

[35] Stuart G.J., Spruston N. (2015). Dendritic integration: 60 years of progress. Nat. Neurosci..

[36] Cui Z., Feng R., Jacobs S., Duan Y., Wang H., Cao X., Tsien J.Z. (2013). Increased nr2a:Nr2b ratio compresses long-term depression range and constrains long-term memory. Sci. Rep..

[37] Bliss T.V., Gardner-Medwin A.R. (1973). Long-lasting potentiation of synaptic transmission in the dentate area of the unanaestetized rabbit following stimulation of the perforant path. J. Physiol..

[38] Engert F., Bonhoeffer T. (1999). Dendritic spine changes associated with hippocampal long-term synaptic plasticity. Nature.

[39] Maletic-Savatic M., Malinow R., Svoboda K. (1999). Rapid dendritic morphogenesis in ca1 hippocampal dendrites induced by synaptic activity. Science.

[40] Ultanir S.K., Kim J.E., Hall B.J., Deerinck T., Ellisman M., Ghosh A. (2007). Regulation of spine morphology and spine density by nmda receptor signaling in vivo. Proc. Natl. Acad. Sci. USA.

[41] Riedel G., Platt B., Micheau J. (2003). Glutamate receptor function in learning and memory. Behav. Brain Res..

[42] Gonda X. (2012). Basic pharmacology of nmda receptors. Curr. Pharm. Des..

[43] Shi L., Adams M.M., Long A., Carter C.C., Bennett C., Sonntag W.E., Nicolle M.M., Robbins M., D’Agostino R., Brunso-Bechtold J.K. (2006). Spatial learning and memory deficits after whole-brain irradiation are associated with changes in nmda receptor subunits in the hippocampus. Radiat. Res..

[44] Hardingham G.E., Fukunaga Y., Bading H. (2002). Extrasynaptic nmdars oppose synaptic nmdars by triggering creb shut-off and cell death pathways. Nat. Neurosci..

[45] Hardingham G.E., Bading H. (2003). The yin and yang of nmda receptor signalling. Trends Neurosci..

[46] Jovanovic J.N., Benfenati F., Siow Y.L., Sihra T.S., Sanghera J.S., Pelech S.L., Greengard P., Czernik A.J. (1996). Neurotrophins stimulate phosphorylation of synapsin i by map kinase and regulate synapsin i-actin interactions. Proc. Natl. Acad. Sci. USA.

[47] Tartaglia N., Du J., Tyler W.J., Neale E., Pozzo-Miller L., Lu B. (2001). Protein synthesis-dependent and -independent regulation of hippocampal synapses by brain-derived neurotrophic factor. J. Biol. Chem..

[48] Yudowski G.A., Olsen O., Adesnik H., Marek K.W., Bredt D.S. (2013). Acute inactivation of PSD-95 destabilizes AMPA receptors at hippocampal synapses. PLoS ONE.

[49] Schluter O.M., Xu W., Malenka R.C. (2006). Alternative n-terminal domains of PSD-95 and SAP97 govern activity-dependent regulation of synaptic AMPA receptor function. Neuron.

[50] Parihar V.K., Pasha J., Tran K.K., Craver B.M., Acharya M.M., Limoli C.L. (2015). Persistent changes in neuronal structure and synaptic plasticity caused by proton irradiation. Brain Struct. Funct..

[51] Leger M., Quiedeville A., Bouet V., Haelewyn B., Boulouard M., Schumann-Bard P., Freret T. (2013). Object recognition test in mice. Nat. Protoc..

[52] Groves T.R., Wang J., Boerma M., Allen A.R. (2017). Assessment of hippocampal dendritic complexity in aged mice using the Golgi-cox method. J. Vis. Exp. JoVE.

[53] Mikolaenko I., Rao L.M., Roberts R.C., Kolb B., Jinnah H.A. (2005). A Golgi study of neuronal architecture in a genetic mouse model for lesch-nyhan disease. Neurobiol. Dis..

[54] Sholl D.A. (1953). Dendritic organization in the neurons of the visual and motor cortices of the cat. J. Anat..

[55] Titus A.D., Shankaranarayana Rao B.S., Harsha H.N., Ramkumar K., Srikumar B.N., Singh S.B., Chattarji S., Raju T.R. (2007). Hypobaric hypoxia-induced dendritic atrophy of hippocampal neurons is associated with cognitive impairment in adult rats. Neuroscience.

[56] Magarinos A.M., Li C.J., Gal Toth J., Bath K.G., Jing D., Lee F.S., McEwen B.S. (2011). Effect of brain-derived neurotrophic factor haploinsufficiency on stress-induced remodeling of hippocampal neurons. Hippocampus.

